# Extracellular *Paracoccidioides brasiliensis *phospholipase B involvement in alveolar macrophage interaction

**DOI:** 10.1186/1471-2180-10-241

**Published:** 2010-09-15

**Authors:** Deyze Alencar Soares, Rosângela Vieira de Andrade, Simoneide Sousa Silva, Anamélia Lorenzetti Bocca, Sueli Maria Soares Felipe, Silvana Petrofeza

**Affiliations:** 1 Instituto de Ciências Biológicas, Universidade Federal de Goiás, 74.001-940 Goiânia, GO, Brazil; 2 Programa de Pós-graduação em Ciências Genômicas e Biotecnologia, Universidade Católica de Brasília, 70790-160, Brasília, DF, Brazil; 3 Departamento de Biologia Celular, Universidade de Brasília, 70910-900, Brasília, DF, Brazil

## Abstract

**Background:**

Phospholipase B (PLB) has been reported to be one of the virulence factors for human pathogenic fungi and has also been described as necessary for the early events in infection. Based on these data, we investigated the role of PLB in virulence and modulation of the alveolar pulmonary immune response during infection using an *in-vitro *model of host-pathogen interaction, i.e. *Paracoccidioides brasiliensis *yeast cells infecting alveolar macrophage (MH-S) cells.

**Results:**

The effect of PLB was analyzed using the specific inhibitor alexidine dihydrochloride (0.25 μM), and pulmonary surfactant (100 μg mL^-1^), during 6 hours of co-cultivation of *P. brasiliensis *and MH-S cells. Alexidine dihydrochloride inhibited PLB activity by 66% and significantly decreased the adhesion and internalization of yeast cells by MH-S cells. Genes involved in phagocytosis (*trl2*, *cd14*) and the inflammatory response (*nfkb, tnf-α, il-1β*) were down-regulated in the presence of this PLB inhibitor. In contrast, PLB activity and internalization of yeast cells significantly increased in the presence of pulmonary surfactant; under this condition, genes such as *clec*2 and the pro-inflammatory inhibitor (*nkrf*) were up-regulated. Also, the pulmonary surfactant did not alter cytokine production, while alexidine dihydrochloride decreased the levels of interleukin-10 (IL-10) and increased the levels of IL-12 and tumor necrosis factor-α (TNF-α). In addition, gene expression analysis of *plb1*, s*od3 *and *icl1 *suggests that *P. brasiliensis *gene re-programming is effective in facilitating adaptation to this inhospitable environment, which mimics the lung-environment interaction.

**Conclusion:**

*P. brasiliensis *PLB activity is involved in the process of adhesion and internalization of yeast cells at the MH-S cell surface and may enhance virulence and subsequent down-regulation of macrophage activation.

## Background

Paracoccidioidomycosis (PCM) is the most prevalent systemic mycosis in Latin America. Epidemiological data indicate a broad geographic distribution in Central and South America, from Mexico to Argentina [1]. It is estimated that as many as ten million individuals may be infected with *P. brasiliensis *in this part of the world. Infection occurs primarily in the lungs, from where it can disseminate via the bloodstream and/or lymphatic system to many organ systems, resulting in the disseminated form of PCM [2].

Considering the pathogenesis of this disease, the initial stages are of importance since this is when resident pulmonary macrophages interact with the fungus for the first time and become activated.

In this context, multiple characteristics have been proposed as virulence factors that enable the invading organism to cause disseminated infections in susceptible hosts. The ability to recognize and adhere to host tissues, to respond rapidly to changes in the external environment, and to secrete enzymes are all thought to play important roles in virulence. Secretion of enzymes, such as phospholipases, has been proposed as one of the strategies used by bacteria, parasites, and pathogenic fungi for invasion of the host and establishment of infection [3].

The role of extracellular phospholipases, particularly phospholipase B (PLB), as potential virulence factors for pathogenic fungi, including *Candida albicans *[4,5]*, Cryptococcus neoformans *[6-10], and *Aspergillus fumigatus *[11] has been reported, although the underlying mechanism has yet to be elucidated. Extracellular phospholipase activities have also been detected in *in-vitro *cultures of *P. brasiliensis *[12], and PLB has been postulated as a potential virulence factor for this pathogen by *in-silico *analysis [13].

Phospholipases are ubiquitous enzymes that are involved in a wide range of biological functions, such as membrane homeostasis, nutrient acquisition, and generation of bioactive molecules. These enzymes are known to contribute to bacterial and fungal virulence through a variety of different interactions with eukaryotic host cells, [14] and to modulate the innate and acquired immune response of the host by generating second messengers such as diacylglycerol or the eicosanoid precursor arachidonic acid [15]. Furthermore, phospholipase-mediated IL-8 release induces the host inflammatory response [14].

It has been shown that secreted PLB1, a proven virulence determinant of *C. neoformans*, is required for the initiation of interstitial pulmonary cryptococcosis, being important for the binding of this fungus to human lung epithelial cells prior to its internalization [9]. PLB1, the product of the CnPLB1 gene, is a multifunctional enzyme which can degrade dipalmitoylphosphatidylcholine (DPPC), the main component of lung surfactant [7].

The goal of this work was to determine whether *P. brasiliensis *PLB is involved in adhesion of this fungus to and internalization by alveolar macrophage (MH-S) cells. Also, we investigated the role of this enzyme in virulence and modulation of the alveolar pulmonary immune response during infection using alexidine dihydrochloride as a specific PLB inhibitor, as well as pulmonary surfactant (Survanta) as a substrate rich in phospholipids.

## Results and discussion

The first contact between *P.brasiliensis *and the host occurs by inhalation of the infectious propagules from the environment. PLB has been reported as a potential virulence factor by transcriptome analysis in *P. brasiliensis *[13,16]. Furthermore, experiments performed by our group showed that the *plb1 *gene is up-regulated during the early events of murine pulmonary infection in a paracoccidioidomycosis model (data not shown), suggesting a possible role for this enzyme in the host-pathogen interaction and reinforcing the hypothesis that PLB could be an important virulence factor for *P. brasiliensis*.

In *C. neoformans*, PLB is necessary for the early events of pulmonary infection and for dissemination from the lung via the lymphatic system and blood [9,17]. Specifically, adhesion to alveolar macrophage cells is reduced in a PLB deletion mutant of *C. neoformans *and also in the presence of selective chemical inhibitors of PLB and a specific anti-PLB antibody. The extent of adhesion was correlated with PLB activity, but not with lysophospholipase (LPL) or lysophospholipase transacylase (LPTA) activity [9].

Lack of established protocols for conducting experiments that might lead to gene disruption or silencing in *P. brasiliensis *hinders the validation of the *plb *gene functionality in this pathogen. In view of this fact, we decided to investigate the role of PLB using an *in-vitro *model of host-pathogen interaction, i.e. the yeast cells of *P. brasiliensis *infecting MH-S cells. The use of a specific inhibitor and/or an activator of PLB could be an effective strategy for investigating the possible role of this enzyme during host-pathogen interaction.

### Effects of alexidine dihydrochloride and pulmonary surfactant on cell viability, adhesion, internalization, and PLB activity during co-cultivation of P. brasiliensis and MH-S cells

**Figure 1:**
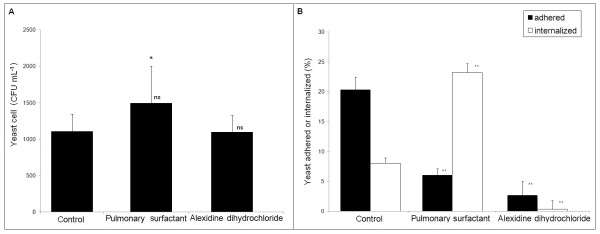
***Paracoccidioides brasiliensis *isolate Pb18 yeast cell viability and infection index after co-culture with alveolar macrophage (MH-S) cells**. (A) CFU of *P. brasiliensis *isolate Pb18 yeast cells; (B) Infection index of *in-vitro *MH-S cells in the presence of alexidine dihydrochloride (0.25 μM) and pulmonary surfactant (100 μg.mL^-1^). Percentage of MH-S cells infected with *P. brasiliensis *yeast cells - adhered (black bar) or internalized (white bar). In all experiments, MH-S cells and opsonized yeast cells were incubated at a yeast-to-macrophage ratio of 1:5, at 37°C in an atmosphere of 5% CO_2 _as described in the Materials and Methods. Data shown are derived from two *in-vitro *independent experiments performed in triplicate (mean ± SEM, with *significance assumed in the range of P < 0:05); ns = non-significantly (P < 0.05); **Significantly different from the untreated control P < 0.001 by the paired 2-tailed Student's *t*-test.

In order to verify whether the treatment with alexidine dihydrochloride and pulmonary surfactant interferes with cell viability, colony-forming unit (CFU) analysis was performed after co-cultivation of *P. brasiliensis *and MH-S cells. Cell viability of *P. brasiliensis *was evaluated by CFU analysis after treatment with the PLB inhibitor (0.25 μM alexidine dihydrochloride) and 100 μg mL^-1 ^pulmonary surfactant. The percentage of cell viability was not significantly altered 6 h post-infection (Figure 1A).

To further investigate the role of PLB we evaluated the percentage of *P. brasiliensis *yeast cells adhered to or internalized by MH-S cells after pulmonary surfactant and alexidine dihydrochloride treatments. The addition of 100 μg mL^-1 ^pulmonary surfactant increased PLB activity by 28% (Table 1), leading to a strong positive effect on the internalization of *P. brasiliensis *(Figure 1B), at least a 3-fold increase in comparison with the control. Also, the proportion of internalized yeast cells (23%) was higher than the proportion of yeast cells adhered to macrophage surfaces (6%). In contrast, we found that 0.25 μM alexidine dihydrochloride caused an 8-fold inhibition in the levels of phagocytosis by MH-S cells compared with the control (Figure 1B). No effects of alexidine dihydrochloride or pulmonary surfactant on adhesion and internalization of heat-killed *P. brasiliensis *were observed (data not shown).

**Table 1:** Phospholipase B activities secreted under the experimental conditions used for Phagocytic test

Treatment	Specific activity of PLB **(μmol min**^**-1 **^**mg**^**-1 **^**protein)**
Untreated control	1.21 ± 0.02
Pulmonary surfactant (100 μg mL^-1^)	1.55 ± 0.06* (28% activation)
Alexidine dihydrochloride (0.25 μM)	0.41 ± 0.08* (66% inhibition)

Phospholipase B activities were assayed after 6 h of co-cultivation of alveolar macrophage (MH-S) cells with *P. brasiliensis *yeast cells with pulmonary surfactant (100 μg mL^-1^) and alexidine dihydrochloride (0.25 μM), as well as without treatment (untreated control), as described in Materials and Methods. *Significantly different from the untreated control, P < 0.05 by the paired 2-tailed Student's *t*-test. Results are means ± SEM of triplicate assays.

A role for PLB activity in adhesion of *C. neoformans *to lung epithelial cells has already been proposed [9]; DPPC is predicted to be the favored lipid substrate for PLB, leading to the production of glycerophosphocholine and free palmitic acid. In this context, it is hypothesized that the addition of pulmonary surfactant (rich in DPPC) would increase the adhesion of *P. brasiliensis *yeast cells to MH-S cells. These results strongly suggest that PLB activity is important in *P. brasiliensis *adhesion to and/or internalization by MH-S cells.

In the present study, enzyme activities were tested under conditions used for adhesion (Table 1). *P. brasiliensis *produced high levels of PLB at 6 h post-infection. 0.25 μM Alexidine dihydrochloride selectively inhibited PLB activity by 66%. In contrast, PLB activity in the presence of 100 μg mL^-1 ^pulmonary surfactant was significantly increased (28%) compared to the control experiment.

### Modulation of P. brasiliensis and MH-S genes in the host-pathogen interaction

**Figure 2:**
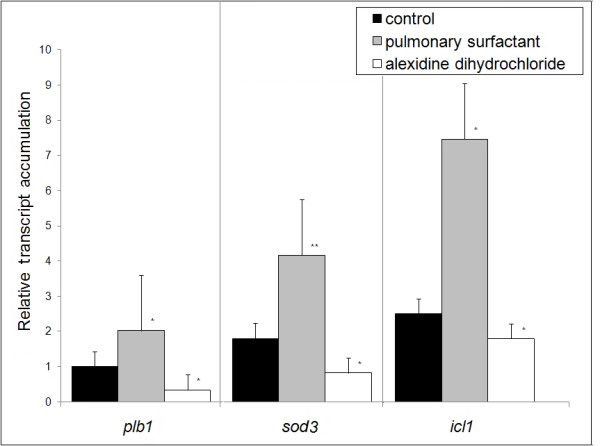
**Real-Time RT-PCR**. Analysis of the transcript level of *Paracoccidioides brasiliensis *genes related to oxidative stress - superoxide dismutase (*sod3*); metabolism - isocitrate lyase (*icl1*) and hydrolytic enzyme phospholipase B (*plb1*). The assay was carried out in triplicate (mean ± SEM); Significantly different from controls: (*P < 0:05 and **P < 0:001) by the paired 2-tailed Student's *t*-test.

Real-time quantitative reverse-rranscriptase-polymerase chain reaction (qRT-PCR) analysis confirmed that the *plb1 *(PLB)*, sod3 *(Cu, Zn superoxide dismutase - SOD), and *icl1 *(isocitrate lyase) genes were up-regulated in *P. brasiliensis *yeast cells during 6 h of interaction with MH-S cells in the presence of pulmonary surfactant. The *sod*3 gene presented a 4.1-fold increase in expression (Figure 2) and under these conditions a higher percentage of yeast cell internalization was observed (Figure 1B). In addition, the i*cl1 *and *plb1 *genes presented 7.4-fold and 2-fold increases in their transcripts levels, respectively. However, in the presence of alexidine dihydrochloride, the levels of transcripts strongly decreased, reaching 0.3-, 0.8- and 1.8-fold for *plb1, sod *3, and *icl1*, respectively (Figure 2).

*P. brasiliensis *metabolic adaptation in response to phagocytosis involves the induction of *sod*3, which encodes a putative Cu, Zn SOD, an enzyme participating in the elimination of superoxide anions. *In-silico *analysis showed that *P. brasiliensis sod*3 corresponds to a putative membrane-bound, glycosylphosphatidylinisotol (GPI)-anchored Cu, Zn SOD, which would allow for better accessibility to host-derived superoxide anions and subsequent rapid detoxification of reactive oxygen intermediates (ROI) [18,19]. The up-regulation of *sod*3 expression in *P. brasiliensis *internalized by pulmonary surfactant-treated MH-S cells provides evidence that *sod*3 may also be needed for the elimination of generated superoxides, thus increasing yeast cell survival. This suggests that the *sod3 *gene is probably involved in the survival of *P. brasiliensis*, corroborating previous data [18].

Induction of the glyoxylate cycle upon phagocytosis has been described as an important adaptation by pathogens to the glucose-poor environment within macrophages, since it facilitates the assimilation of two-carbon compounds, the product of fatty acid degradation [20,21]. In *P. brasiliensis*, both isocitrate lyase and the entire glyoxylate pathway have been shown to be enhanced under low glucose and oxygen tension, in the presence of acetate and high temperature, as well as during intracellular growth [16,22,23]. Our results showed that the *icl1 *gene was up-regulated under increased PLB activity, which could be correlated with the fungal survival inside macrophage cells.

The results observed for the gene expression of *plb1*, s*od3*, and *icl1 *suggest that, under *in-vitro *conditions mimicking the lung-environment interaction, gene re-programming was similar to that described for peritoneal macrophages [18,24], corroborating the importance and effective participation of those genes in the process of adaptation by the fungus to this inhospitable environment.

The process of recognition of pathogen-associated molecular patterns (PAMP) depends on the pattern recognition receptors (PRR) present in great diversity in the plasma membrane of phagocytes [25]. The two main members of this family that recognize fungal components are the C-type lectin-like receptors (CLRs) and toll-like receptors (TLRs) [26].

To investigate whether *P. brasiliensis *PLB is able to affect the inflammatory response of MH-S cells, we assessed the transcription level of the following key genes: *tnf-α *(tumor necrosis factor-alpha), *il-1β *(Interleukin-1β), *nkrf *(NFKappaB repressing factor), and *nfkb *(P50 subunit of NFKappaB), known to be involved in the phagocytic process, and *trl2 *(toll-like receptor 2), *cd14 *(glycosyl-phosphatidylinositol-anchored glycoprotein), and *clec*2 (C-type lectin-like receptor), signal receptors involved in controlling the immune response. These genes had already been reported to be differentially expressed by peritoneal macrophages infected with *P. brasiliensis *[24].

**Figure 3:**
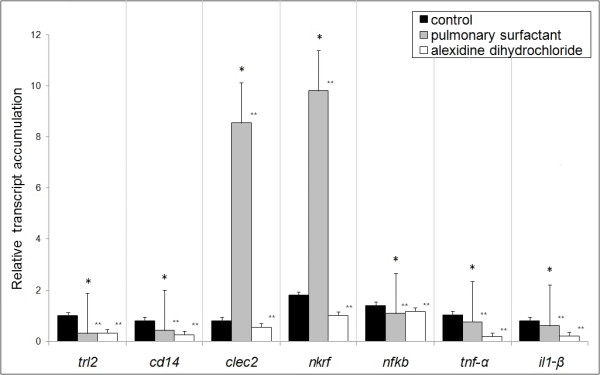
**Real-Time RT-PCR**. Analysis of the transcript level of macrophage genes related to phagocytosis (*clec2*, *trl2*, and *cd14*) and inflammation (*nkrf*, *nfkb*, *tnf-α*, and *il-1β*). The assay was carried out in triplicate (mean ± SEM, with *significance assumed in the range of P < 0:05); **Significantly different from controls: P < 0.001 by the paired 2-tailed Student's *t*-test.

In our experiments, *trl2, cd14, Il-1β, nfkb*, and *tnf-α *genes, which play an important role in the host innate response, were down-regulated during *P. brasiliensis*-MH-S cell interaction in the presence of pulmonary surfactant or alexidine dihydrochloride compared to the control (Figure 3). In contrast, the main up-regulated genes were those encoding the membrane-related protein CLEC 2 (*clec2*) - a mannose-type receptor, important for more effective phagocytic capacity [27] - and the pro-inflammatory inhibitor (*nkrf*), presenting fold-changes of 8.0 and 9.8 respectively, in cultures exposed to the pulmonary surfactant (Figure 3).

NFkB is a key transcription factor involved in TLR-mediated innate immunity and together with its repressor Nkrf is an important regulator of the inflammatory process, a powerful protective mechanism coordinated and controlled by cytokines and chemokines. Our data showed an up-regulation of the *nkrf *gene in the presence of the pulmonary surfactant, suggesting a possible modulation of the innate immune response under conditions of increased PLB activity.

### Cytokine production by MH-S cells during host-pathogen interaction

**Figure 4:**
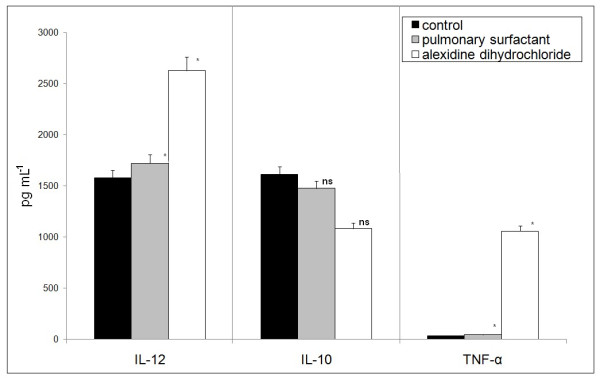
**Amount of cytokines and tumor necrosis factor-α released by alveolar macrophage (MH-S) cells infected with *Paracoccidioides brasiliensis***. The assay was carried out in triplicate (mean ± SEM); ns = non-significantly and *significantly different from controls: P < 0.05 by the paired 2-tailed Student's *t*-test.

In order to verify the pattern of MH-S cell activation, the levels of the cytokines interleukin-10 (IL-10), IL-12, and tumor necrosis factor-α (TNF-α) were determined. When compared to the control, the MH-S cells treated with alexidine produced higher levels of IL-12 and TNF-α and lower levels of IL-10. However, no significant difference between the control group and the group treated with surfactant was observed (Figure 4).

In the course of experimental fungus infection, cell-mediated immunity is critical for host defense [28]. The successful resolution of *P. brasiliensis *infection depends on a strong Th1 immune response and down-regulation of Th2 cytokine production. The immune response involving a preferential Th1 activation, with IFN-γ production and efficient macrophage activation, is able to control fungal dissemination. IFN-γ production is partly dependent on IL-12 production in macrophages [29].

Our results demonstrated that the interaction between MH-S and yeast cells, in the presence of PLB, is capable of shaping macrophage activation, compromising the induction of the Th1 response and strongly suggesting a pathogen evasion mechanism.

**Figure 5:**
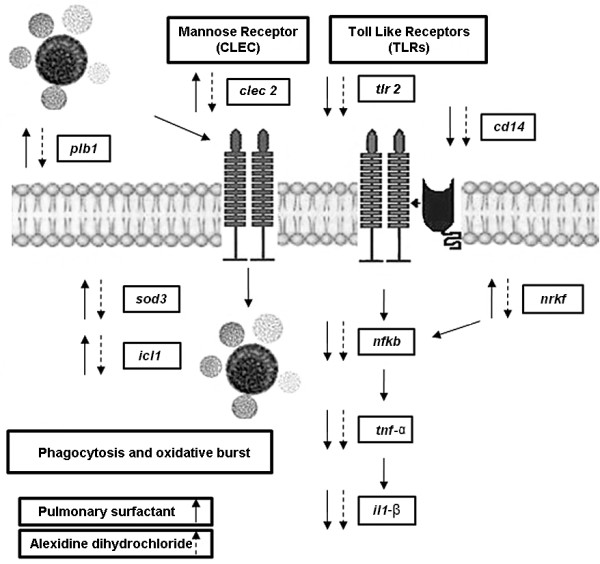
**Model of expression differential genes in presence of the surfactant and alexidine, respectively**. The small arrows indicate induced **(↑) **and repressed **(↓) **genes. *Paracoccidioides brasiliensis *survival in macrophage phagosome and burst oxidative: *plb1*, *icl1*, and *sod3*. Macrophage genes: *clec2*, *trl2*, *cd14*, *nfkb, nkrf*, *tnf*-α, and *il-1β*.

Based on these results, we propose the model presented in Figure 5 to explain the phagocytic mechanism of the interaction between *P. brasiliensis *and MH-S cells. In the presence of the activator of PLB activity (pulmonary surfactant), a stimulation of the mannose-receptor CLEC signal transduction pathway probably occurs, since expression of this gene is induced. The up-regulated *clec-2 *and *nkrf *and the down-regulated *nfkb, tnf-α*, and *il-1β *genes provide evidence that the mannose-receptor CLEC is the probable mediator of fungal phagocytosis. This is further supported by the increased adherence and internalization of yeast cells by MH-S cells in the presence of the surfactant. Also, the *trl*2 and *cd*14 genes are down-regulated, reinforcing the hypothesis that phagocytosis is probably occurring via the CLEC mannose receptor. In contrast, in the presence of the inhibitor of PLB - alexidine dihydrochloride -, the *clec*2 and *nkrf *genes are repressed, which also corroborates this hypothesis. Furthermore, adhesion and internalization are stimulated and, consequently, a gene expression re-programming occurs regarding the genes involved in the survival of the pathogen inside the MH-S cells.

Fungal PLB exhibits a function related to the regulation of immune responses via the liberation of fatty acid precursors (arachidonic acid, linolenic acid, or eicosanopentaenoic acid) for host eicosanoid synthesis [15]. The production of eicosanoids, potent regulators of host immune responses, including prostaglandins and leukotrienes by fungi in the lungs, may also play a role in modulating the Th1-Th2 balance in the immune response, and may promote eosinophil recruitment or survival of the fungus in the lungs [15]. *In-vivo *and *ex-vivo P. brasiliensis *infection has been recently proven to induce leukotriene synthesis, which could explain the low levels of cytokines IL-10, IL-12, and TNF-α, and confirm a pattern capable of interfering in the host response to the fungus [30]. Thus, in the presence of surfactant there is increased activity of PLB, and probably a greater release of substrates for lipid synthesis and production of leukotrienes, which act as suppressors of the innate immune response, confirming the low expression levels of the cytokine *tnf*-α and *il-1*β genes. In the proposed model, the genes related to phagocytosis and oxidative burst are up-regulated providing an efficient mechanism for fungal survival. The increase in IL-12 and decrease in IL-10 after inhibition of PLB participate in the enhancement of IFN-γ activity, which is capable of inducing a cellular immune response. These data confirm the participation of PLB in the mechanism of fungal evasion, interfering with an adequate immune response by the host.

## Conclusions

Based on these data, we conclude that *P. brasiliensis *PLB is important for adhesion and internalization of yeast cells by MH-S cells. Whether PLB activity results from the production of eicosanoids or leukotrienes or not remains unknown, although studies are in progress to investigate this possibility. Nevertheless, our study clearly identified activities of fungal PLB that may enhance virulence and subsequent down-regulation of macrophage activation.

## Methods

### Strains, cultures and reagents

*P. brasiliensis *Pb18 (ATCC 32069) yeast cells were cultivated in Fava-Netto semisolid medium for 7 days at 37°C and used in *in-vitro *infection. Alveolar macrophage lineage MH-S (ATCC CRL-2019) was grown in RPMI-1640 tissue culture medium (Sigma-Aldrich, Inc., St. Louis, MO, USA) supplemented with 20 mM HEPES, 1.5 g L^-1 ^sodium bicarbonate, 2.5 mg mL^-1 ^gentamicin, and 10 U mL^-1 ^heparin. The viability of MH-S cells was determined by trypan blue exclusion. All assays used the bovine pulmonary surfactant Survanta (Abbott Laboratories, Inc., Columbus, OH, USA), which is an extract of bovine lung containing about 75% DPPC and 45% phosphatidylcholine (PC), generating substrates for phospholipases. The specific inhibitor of PLB - alexidine dihydrochloride (Toronto Research Chemicals, Inc., Toronto, Ontario, Canada) - was prepared as a stock solution at 10 mM in dimethyl sulfoxide (DMSO), which was then diluted to the required concentration with RPMI medium.

### Infection of MH-S cells with P. brasiliensis yeast cells

#### Phagocytic test

MH-S cells were seeded in 24-well (0.2 × 10^5 ^cells/well) or in 150 cm^2 ^(0.4 × 10^7 ^cells/well) cells culture flasks and incubated at 37°C for 6 h. Non-adherent cells were removed by washing, whereas the adherent cells were incubated in RPMI supplemented as stated above, with 10% heat-inactivated fetal calf serum, at 37°C. *P. brasiliensis *yeast cells were suspended in RPMI medium containing 20% fresh mouse serum. The opsonization protocol was carried out by incubation of yeast cell suspension at 37°C for 30 min. MH-S cell monolayers were infected with 4 × 10^6 ^yeast cells, representing a yeast-to-macrophage ratio of 1:5 [31]. Incubation was carried out at 37°C in a humidified 5% CO_2 _atmosphere.

The influence of PLB on the phagocytic indices was evaluated by adding different concentrations of the surfactant (100 μg mL^-1 ^and 200 μg mL^-1^) and alexidine dihydrochloride (0.25 μM and 0.50 μM) to the culture medium at the beginning (T0) of the experiments.

We selected a 6-hour period for infection because it represents an early time point of fungal cell internalization by macrophages [18]. After infection, the culture was fixed with methanol and stained with Wright-Giemsa (Sigma-Aldrich, Inc., St. Louis, MO, USA). *P. brasiliensis *cells were counted in order to evaluate the percentage of attached or internalized yeast cells after infection. Experiments were performed in triplicate, and 12 microscopic fields were assessed. The results are presented as mean ± SEM (standard error of the mean).

### Colony forming unit (CFU) determination

The number of viable fungal cells after phagocytosis by MH-S cells was assessed by CFU counts. MH-S cells were challenged with *P. brasiliensis *yeast cells and incubated for 6 h as described for the phagocytic test. After this time, cultures were rinsed with RPMI to remove non-internalized yeast cells and distilled water was added to lyse the macrophages. The cellular suspension was harvested, washed in phosphate buffered saline (PBS), and the final pellets were resuspended in 1 mL of PBS. Aliquots of 100 μL of each sample were added to agar plates (4% SFB, 5% BHI solid medium) and colonies per plate were counted after 8-10 days of incubation at 37°C.

### RNA extraction

Total RNA from *P. brasiliensis *yeast cells internalized by MH-S cells and RNA from MH-S cells were extracted after 6 h of co-cultivation with pulmonary surfactant (100 μg mL^-1^) and alexidine dihydrochloride (0.25 μM), as well as without treatment (control). Extracellular and weakly adherent fungal cells were removed by washing with pre-warmed RPMI. Macrophages were then lysed with a guanidine thiocyanate-based solution [32] and intact fungal cells were harvested by centrifugation (8000 × *g *for 10 min) immediately followed by Trizol total RNA extraction (Invitrogen Corp., Carlsbad, CA, USA) according to the manufacturer's instructions. Total RNA from *in-vitro *grown *P. brasiliensis *yeast cells and MH-S cells was also extracted with Trizol, to be used as controls.

### Phospholipase B assay

Supernatants were obtained after cell centrifugation at 10000 × *g *for 15 min and assayed for PLB activity using DPPC as a substrate by the radiometric assay method [7]. The carriers, DPPC (800 mM) and 1,2-di [1-^14^C] palmitoyl-phosphatidylcholine (20,000 dpm), were dried under nitrogen and resuspended in 125 mM imidazole-acetate buffer, pH 4.0. The reaction was initiated by adding culture supernatant (1 mg of total protein), and after incubation for 30 min the rate of radiolabeled PC loss was measured.

Reaction products were extracted, separated by thin-layer chromatography (TLC), and quantified. Measurements were repeated in three experiments for each treatment and the data were presented as the average of the three. PLB activity was expressed as mM of substrate hydrolyzed per minute, per milligram of protein. Total protein concentrations were measured using the Protein Assay kit (Quant-iT - Invitrogen Corp., Carlsbad, CA, USA). Significance tests were carried out comparing each treatment with the control value (100%) using a one-sample Student's *t*-test. *P *< 0.05 was taken as the limit to indicate significance.

### Real-time RT-PCR validation of differentially expressed genes

The real-time RT-PCR system using SYBR Green detection (Applied Biosystems) was used to analyze gene expression in RNA samples. After treatment with DNase I (Invitrogen Corp., Carlsbad, CA, USA) in the presence of RNase inhibitor (Invitrogen Corp., Carlsbad, CA, USA), equal amounts of RNA (1 μg) were reverse transcribed using oligo(dT)12-18 primer and submitted to real time PCR. Amplification assays were carried out with a 7900HT Sequence Detection System ABI PRISM instrument (Applied Biosystems, Carlsbad, CA, USA) in 12 μL reactions containing 0.4 μM of each primer (listed in Tables 2 and 3), 6 μL of SYBR Green PCR Master mix (2 ×), and 0.2 μL of template cDNA. After initial denaturation at 95°C for 10 min, amplifications were performed for 40 cycles of: 95°C for 15 s followed by 60°C for 1 min.

**Table 2:** Primers *Paracoccidioides brasiliensis *used for real time RT-PCR

**Cluster ID**^**a**^	**Gene**^**b**^	Forward primer (5'-3')	Reverse primer (5'-3')
50	*sod3*	CTGTTCGCTGGGCTTTGC	TCAGTAGTGACGGCTTCCATCAT
1688	*icl1*	GCTCACCCAGATGGTCAAAT	AGTATCCGCATCCGCAATAA
3306	*plb1*	GCAATGCAAGGGAAGAAAGA	CGATCCGAGGAACTCTAACG

^a ^ID: identification.

^b ^Abbreviations: *sod3 *(Cu, Zn superoxide dismutase); *icl1 *(isocitrate lyase); *plb1 *(phospholipase B).

**Table 3:** Primers for real time RT-PCR to measure gene expression using RNA from alveolar macrophage (MH-S) cells

**Cluster ID**^**a**^	**Gene**^**b**^	Forward primer (5'-3')	Reverse primer (5'-3')
272294	*Rps9*	CGCCAGAAGCTGGGTTTGT	CGAGACGCGACTTCTCGAA
21961	*nkrf*	ACCTTTCAACCTACGATGGTCAGA	GAGCTCTCACATGGAATTTGGAA
575033	*nfkb*	AGCCAGCTTCCGTGTTTGTT	AGGGTTTCGGTTCACTAGTTTCC
104798	*tnf -α*	GTACCTTGTCTACTCCCAGGTTCTCT	GTGGGTGAGGAGCACGTAGTC
574821	*clec*2	CTCTTCTTGGTGGCGTGTGA	AACAACCAGCCCCATGGA
3989461	*il-1β*	GTGTGTGACGTTCCCATTAGACA	CAGCACGAGGCTTTTTTGTTG
1346060	*trl2*	AAGAGGAAGCCCAAGAAAGC	CGATGGAATCGATGATGTTG
5120996	*cd14*	CGCAGCCTGGAATACCTTCTA	CCGCTTTAAGGACAGAGACTTGATA

^a ^ID: identification.

^b ^Abbreviations: *Rps*9 (constitutive ribosomal macrophage gene); *nkrf *(NFKappaB repressing factor); *nfkb *(P50 subunit of NFKappaB); *tnf-α *(tumor necrosis factor-alpha); *clec*2 (C- type lectin like receptor); *il-1β *(Interleukin-1β); *trl2 *(toll-like receptor 2); *cd14 *(glycosyl-phosphatidylinositol-anchored glycoprotein).

The comparative crossing threshold (CT) method, employing the constitutive ribosomal *Rps9 *macrophage gene or *P. brasiliensis *α-tubulin gene, was used in order to normalize the expression value of each gene of interest in the macrophage infected sample compared with the non-infected control. Real time RT-PCR experiments were carried out in triplicate for all analyzed genes.

### Analysis of cytokine secretion by MH-S cells

Supernatants of co-cultured cells from the different treatments, obtained as described above, were used for the detection of cytokine production. The levels of cytokines IL-10, IL-12, and TNF-α were measured using a commercial ELISA kit (BD Biosciences, San Diego, CA, USA) according to the manufacturer's guidelines. The cytokine levels in the supernatant from MH-S cells were calculated based on a standard curve provided with the commercial kit. Data are expressed as mean ± SEM.

### Statistical analysis

Statistical comparisons were performed by the paired 2-tailed Student's *t*-test. All values are reported as mean ± SEM, with significance assumed at p < 0.05.

## Authors' contributions

DAS carried out the co-cultured cell studies, and drafted the manuscript. RVA participated in the transcription analysis experiments and drafted the manuscript. SSS carried out the co-cultured cell experiments. ALB carried out the immunoassays and drafted the manuscript. MSSF participated in the design of the study and drafted the manuscript. SP conceived the study, performed the statistical analysis, participated in its design and coordination. All authors read and approved the final manuscript.
